# The Cerebellum Posterior Lobe Associates with the Exophthalmos of Primary Hyperthyroidism: A Resting-State fMRI Study

**DOI:** 10.1155/2019/8135671

**Published:** 2019-11-28

**Authors:** Wen-Feng Liu, Yong-Qiang Shu, Pei-Wen Zhu, Biao Li, Wen-Qing Shi, Qi Lin, Yu-Xin Liu, Meng-Yao Zhang, You-Lan Min, Qing Yuan, Yi Shao

**Affiliations:** ^1^Department of Ophthalmology, The First Affiliated Hospital of Nanchang University, Nanchang 330006, Jiangxi, China; ^2^Department of Radiology, The First Affiliated Hospital of Nanchang University, Nanchang 330006, Jiangxi, China

## Abstract

**Background:**

Exophthalmos occurs in patients with primary hyperthyroidism. There were few studies about the changes of brain functional networks of patients with exophthalmos of primary hyperthyroidism (EOPH). However, differences in spontaneous brain activity in patients with EOPH remain unclear.

**Objective:**

This study explored alterations in the brain functional networks of patients with EOPH using a voxel-wise degree centrality (DC) method.

**Methods:**

A total of 20 patients with EOPH (8 men and 12 women) were enrolled. In addition, 20 patients with primary hyperthyroidism without exophthalmos, matched in age, sex, and education status, were enrolled as a control group. The Hospital Anxiety and Depression Scale was used to assess the anxiety and depression status of participants. All participants were examined using resting-state functional MRI. Changes in spontaneous brain activity were investigated using the DC method. To distinguish between the DC values of the patients with EOPH and those of the control group, we analyzed the receiver operating characteristic (ROC) curve. The interrelationships between the DC values and clinical variables in the patients with EOPH were evaluated using Pearson's correlation coefficient.

**Results:**

Patients with EOPH exhibited notably lower DC values in the cerebellum posterior lobe than the control group. In addition, there were negative correlations between the anxiety scores (AS) and the depression scores (DS) and DC values of the cerebellum posterior lobe. The ROC curve analysis of the cerebellum posterior lobe demonstrated that the area under the curve method had a high diagnostic accuracy.

**Conclusions:**

Our study was the first, to our knowledge, to explore changes in the brains of patients with EOPH using the DC method. The DC value was significantly different in the cerebellum posterior lobe in patients with EOPH, indicating that the cerebellum posterior lobe is associated with EOPH.

## 1. Introduction

**Figure 1:**
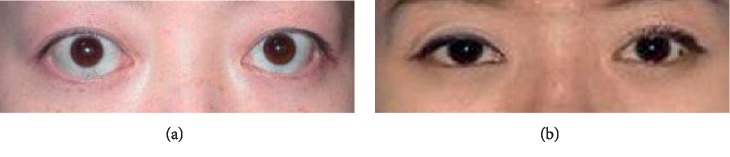
Primary hyperthyroidism patients with exophthalmos (a) and without exophthalmos (b).

Primary hyperthyroidism is a common clinical syndrome, which is caused by excessive synthesis and release of thyroid hormones. Moreover, patients usually experience high metabolic and sympathetic excitation [1, 2]. Many patients with primary hyperthyroidism will develop typical eye symptoms, including exophthalmos, eyelid edema, and visual loss, of which exophthalmos is one of the most significant and specific signs [3, 4].  Figure 1 shows exophthalmos and nonprominent eyes in patients with primary hyperthyroidism.

Exophthalmos of primary hyperthyroidism (EOPH) is characterized by extraocular muscle hypertrophy, an increase in muscle tension, and ocular movement disorder [5]. The ocular protrusion of EOPH is greater than 18 mm, and EOPH often progresses bilaterally [6]. Because patients with EOPH cannot close their eyelids, the conjunctiva and cornea are exposed to air, easily leading to conjunctivitis and keratitis, and even blindness. Previous studies have shown that EOPH might be associated with extraocular muscle hypertrophy, retrobulbar tissue increase, and the invasion of lymphocytes [7]. However, rarely research had focused on the intrinsic brain activity variations of EOPH. The function and contribution of brain tissue are not yet fully determined. Fortunately, brain image scanning might promote noninvasive exploration of alterations in the brain functional networks of patients with EOPH.

In recent years, the development of neuroimaging technology has provided more possibilities for studying the mechanism of neuropsychiatry-related diseases. At the same time, the rapid development of fMRI has made it possible to provide solid evidence that changes in brain regions are closely related to several ophthalmological diseases. Based on the different magnetic properties of oxyhemoglobin and deoxyhemoglobin, blood-oxygen-level dependent signals of different brain regions are obtained, while subjects remain relaxed and awake with closed eyes [8, 9]. fMRI can be used to indirectly search for specific brain activation areas of subjects and is a useful tool in determining the functional connectivity of the human brain. Moreover, the fMRI data can also serve as the physiological basis for information processing and mental representation.

Voxel-wise degree centrality is one method of resting-state fMRI, which measures the topological structure of brain functional connectors at the voxel level and detects the functional relationships between a brain region and the rest of the brain [10]. The DC value represents the number of direct connections of a given voxel in the functional connector, and a high DC value represents a node with substantial direct connections to other nodes [11]. Thus, DC values can objectively and comprehensively reflect the abnormalities of brain functional areas. Therefore, DC is a better network measurement than other methods. Moreover, DC has been utilized to assess the altered brain activity in patients with epilepsy, Parkinson's disease, type-two diabetes, attention deficit hyperactivity disorder, and Alzheimer's disease [12–16]. Accordingly, the DC method is a dependable rs-fMRI technology, which has not yet been used in the study of EOPH. In this study, the DC method is used to further understand converted spontaneous brain activities in patients with EOPH in comparison with patients with primary hyperthyroidism without exophthalmos.

## 2. Materials and Methods

### 2.1. Subjects

A total of 20 patients with EOPH (8 men and 12 women) were recruited from the First Affiliated Hospital of Nanchang University, China. Inclusion criteria were as follows: (1) met the diagnostic criteria for primary hyperthyroidism; (2) exophthalmos confirmed by imaging examination; (3) bilateral exophthalmos; (4) men were 18–60 years old, while women were 18–50 years old; (5) right-handedness. Exclusion criteria were as follows: (1) a history of severe craniocerebral trauma; (2) a history of epileptic seizures, schizophrenia, bipolar disorder, or other mental disorders; (3) a history of alcohol or drug abuse; (4) other endocrine diseases and autoimmune diseases besides hyperthyroidism; (5) pregnancy; (6) claustrophobia or other contraindications for magnetic resonance imaging.

Twenty patients with primary hyperthyroidism without exophthalmos (8 men and 12 women), matched for age, sex, and education status, were also recruited as control groups for this research. Inclusion criteria were as follows: (1) primary hyperthyroidism, (2) no symptoms of exophthalmos, (3) no other endocrine diseases or autoimmune diseases, (4) no history of alcohol or drug abuse, (5) no history of neurological or psychiatric disorders, (6) not pregnant, (7) right-handedness, and (8) no MRI scanning contraindications.

The study received approval from the Medical Ethics Committee of the First Affiliated Hospital of Nanchang University and abided by the Declaration of Helsinki. All participants were informed of the objectives, contents, potential risks, and signed informed consent forms.

### 2.2. Parameters for MRI

A 3-Tesla MR scanner (Trio, Siemens, Munich, Germany) was used for MRI scanning. We acquired whole-brain *T*_1_ weights by using a spoiled gradient-recalled echo sequence with the following parameters: repetition time, 1900 ms; echo time, 2.26 ms; flip angle, 9°; thickness, 1.0 mm; field of view, 250 mm × 250 mm; gap, 0.5 mm; and acquisition matrix, 256 × 256. The scanning parameters were as follows: repetition time, 2000 ms; echo time, 30 ms; flip angle, 90°; thickness, 4.0 mm; field of view, 220 mm × 220 mm; gap, 1.2 mm; acquisition matrix, 64 × 64; 29 axial slices; and 240 volumes.

### 2.3. Data Processing for fMRI

MRIcro (http://www.MRIcro.com), Statistical Parametric Mapping software (SPM8, http://www.fil.ion.ucl.ac.uk/spm), the Data Processing Assistant for rs-fMRI advanced edition (DPARSFA, http://rfmri.org/DPARSF) software, and the Resting-State Data Analysis Toolkit (REST, http://www.restfmri.net) were utilized to prefilter the data. The procedure of preprocessing included the following steps. Firstly, the first 10 volumes of each field are abandoned to achieve balance of the signal and adaptation of the participants. Secondly, the translation (in millimeters) and rotation (in degrees) of each subject is estimated after head movement correction, and volumes with movements in the *x*, *y*, or *z* direction greater than 2 mm are excluded. Then, fMRI images were detrended and bandpass filtered (0.01–0.08 Hz) to reduce effects of low-frequency drift and physiological high-frequency respiratory and cardiac noise. Then, the fMRI images are standardized to the Montreal Neurological Institute (MNI) using standard echo planar imaging templates and resampling at a resolution of 3 mm × 3 mm × 3 mm.

### 2.4. Degree Centrality

On the basis of the individual voxel-wise functional network, we calculated the DC value by counting the number of degrees of the binarized adjacency matrix or significant suprathreshold correlations between the subjects and used the following equation to convert the voxel DC diagram of each individual to a *z*-score map:

(1) $$\begin{array}{c} z_{i}=\text{DC}i-\left(\frac{{\text{mean}}_{\text{all}}}{{\text{std}}_{\text{all}}}\right), \end{array}$$

where *z*_*i*_ refers to the *z*-score of the *i*th voxel, DC_*i*_ is the DC value of the *i*th voxel, and std_all_ refers to the standard deviation [17].

### 2.5. Correlation Analysis

Graphpad prism 7 (GraphPad Software Inc., San Diego, CA, USA) was then used to analyze the correlation between DC mean values in altered brain areas and the clinical behaviors (*p* < 0.05 was taken to indicate significant difference).

### 2.6. Statistical Analysis

The demographic and clinical variables between the EOPH and control groups were compared using SPSS20.0 software (SPSS, Chicago, IL, USA) with the independent samples *t-*test. And *p* < 0.05 was regarded as statistically significant. The statistical threshold of the voxel level for multiple comparisons according to the Gaussian random field (GRF) theory was set at a level of *p* < 0.05. The general linear model analysis generated by the SPM8 toolkit was used to investigate the difference in DC values between patients with EOPH and the control group. The receiver operating characteristic (ROC) curve method was used to distinguish the mean DC values in brain areas of patients with EOPH from those of control groups. The correlations between the DC values of cerebrum areas and the clinical features in patients with EOPH were investigated using Pearson's correlation coefficient.

## 3. Results

### 3.1. Demographics and Behavioral Results

Twenty patients with EOPH (8 men and 12 women) and 20 control group patients (8 men and 12 women) were involved in this study. There was no statistically significant difference in patients' age, sex, or weight or in intraocular pressure between the two groups. However, significant differences existed in the duration of hyperthyroidism (*p*=0.046), the best-corrected right-eye visual acuity (*p*=0.009), the best-corrected left-eye visual acuity (*p*=0.012), and the anxiety (*p* < 0.001) and depression scores (*p* < 0.001) in the two groups. Details are shown in Table 1.

### 3.2. Difference in DC

**Figure 2:**
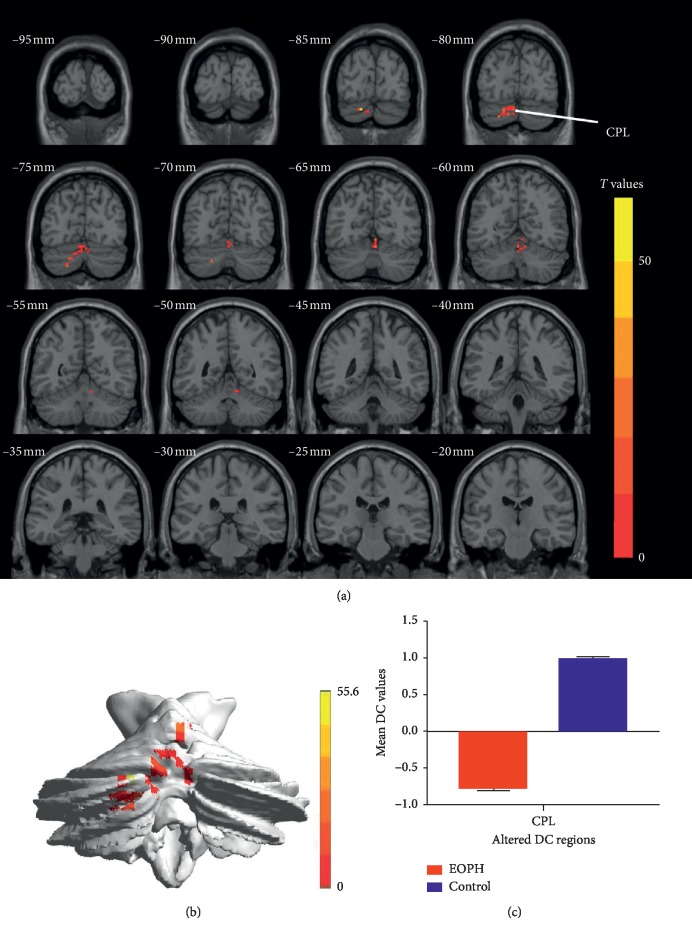
Comparison of DC in the EOPH group and control group. (a) Significant difference in mean DC values between EOPH and control group was recognized in the CPL. (b) The stereoscopic form of the cerebrum. The red regions indicated lower DC values. AlphaSim corrected at a cluster size >40 voxels and a level of *p* < 0.01 for multiple comparisons using Gaussian random field theory. (c) The mean DC values between the EOPH group and control group. DC, degree centrality; EOPH, exophthalmos of primary hyperthyroidism; CPL, cerebellum posterior lobe.

The DC value clearly decreased in the cerebellum posterior lobe in the EOPH group (Figures 2(a) and 2(b) and Table 2). The mean DC between the two groups is shown in Figure 2(c).

### 3.3. Receiver Operating Characteristic (ROC) Curve

**Figure 3:**
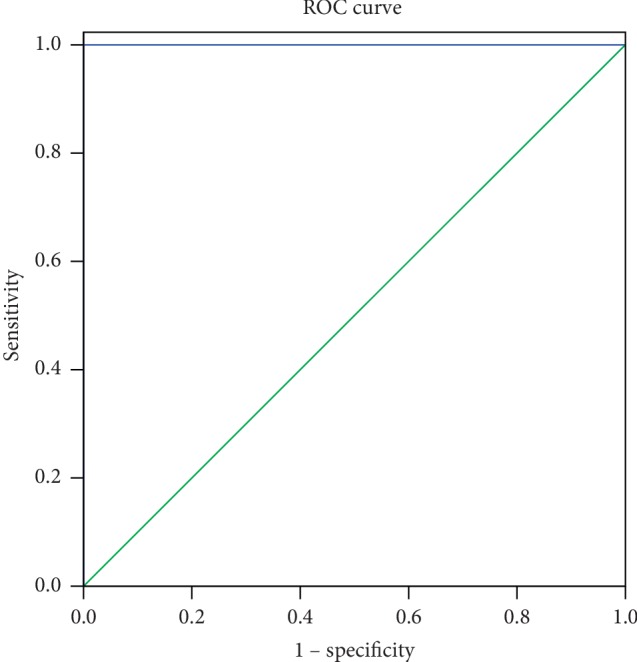
ROC curve analysis of the mean DC values in the cerebellum posterior lobe. The area under the ROC curve was 1.000 (*p* < 0.001; 95% CI: 1.00–1.00) for CPL. ROC, receiver operating characteristic; DC, degree centrality; EOPHs, exophthalmos of primary hyperthyroidism groups.

In this study, the DC values of the cerebellum posterior lobe of the EOPH and control groups are different. And the area under the curve (AUC) of the cerebellum posterior lobe was 1.000 (Figure 3). The area under the ROC curve represents the diagnostic rate: AUC values of 0.5–0.7 indicate that the diagnostic value is limited, AUC values between 0.7 and 0.9 indicate a perfect diagnostic value, and AUC values greater than 0.9 indicate high accuracy.

### 3.4. Correlation Analysis

**Figure 4:**
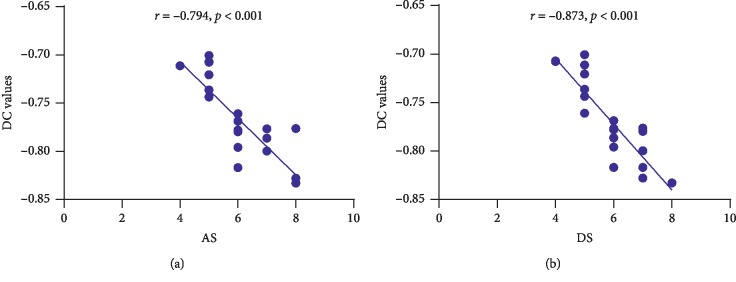
Correlations between the mean DC values of the cerebellum posterior lobe and the clinical behaviors. (a) The AS showed a negative correlation with the DC values of the cerebellum posterior lobe (*r* = −0.794, *p* < 0.001), and (b) the DS showed a negative correlation with the DC values of the cerebellum posterior lobe (*r* = −0.873, *p* < 0.001). DC, degree centrality; AS, anxiety scores; DS, depression scores.

In the EOPH group, anxiety scores negatively correlated with the DC values of the cerebellum posterior lobe (*r* = −0.794, *p* < 0.001); depression scores also negatively correlated with the DC values of the cerebellum posterior lobe (*r* = −0.873, *p* < 0.001) (Figure 4).

## 4. Discussion

**Figure 5:**
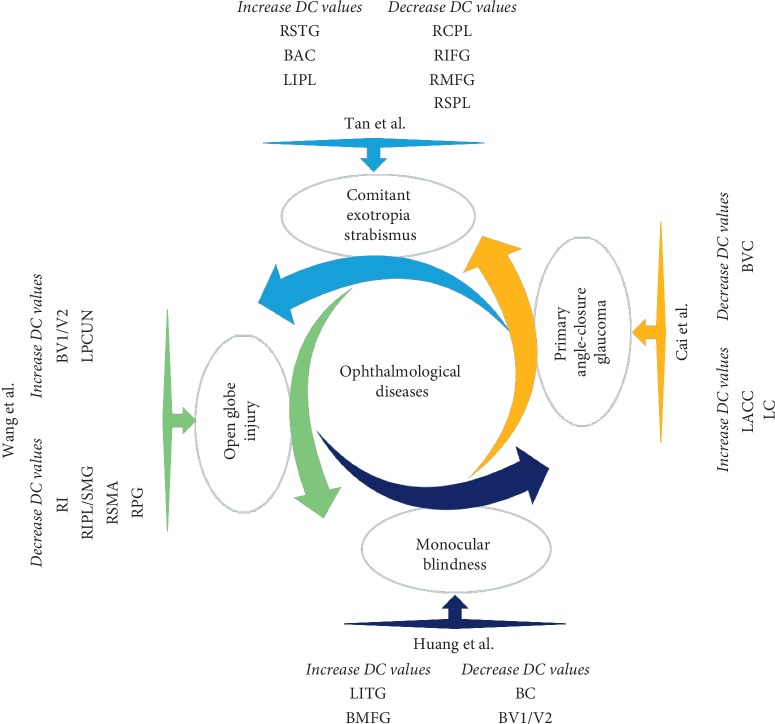
DC method applied in ophthalmological diseases. DC, degree centrality; RSTG, right superior temporal gyrus; BAC, bilateral anterior cingulate; LIPL, left inferior parietal lobule; RCPL, right cerebellum posterior lobe; RIFG, right inferior frontal gyrus; RMFG, right middle frontal gyrus; RSPL, right superior parietal lobule; LACC, left anterior cingulate cortex; LC, left cuneus; BVC, bilateral visual cortices; LITG, left inferior temporal gyrus; BMFG, bilateral medial frontal gyrus; BC, bilateral cuneus; BV1/V2, bilateral primary visual cortex; LPCUN, left precuneus; RI, right insula; RIPL/SMG, right inferior parietal lobule/supramarginal gyrus; RSMA, right supplementary motor area; RPG, right postcentral gyrus.

Resting-state fMRI can effectively display spatial patterns of temporal correlation. The DC method is a new and reliable rs-fMRI method for revealing changes in brain functional connectivity and detecting and quantifying activation sites in the brain. This method has already been applied effectively in the study of various ocular diseases, including glaucoma, comitant exotropia strabismus, late monocular blindness, and open globe injury (Figure 5) [18–21]. As far as we know, this is the first time that the DC method has been used to identify changes in the endogenous functional connectivity on the basis of voxel-based global brain correlation analysis in individuals with EOPH.

**Figure 6:**
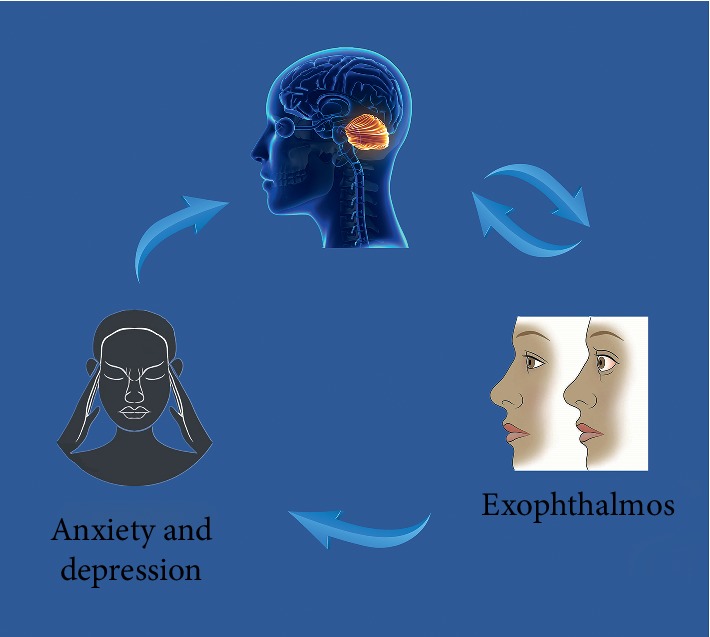
Correlations between mean DC signal values and behavioral performance. Compared with the control group, the DC value of the CPL was decreased in EOPH patients. And the EOPH patients exhibited with more anxiety and depression. DC, degree centrality; CPL, cerebellum posterior lobe; EOPH, exophthalmos of primary hyperthyroidism.

In this study, we demonstrated altered intrinsic brain connectivity patterns of the cerebellum posterior lobe in individuals with EOPH. The EOPH group exhibited significantly decreased DC signal values in the cerebellum posterior lobe. In addition, anxiety and depression scores were negatively correlated with DC values of the cerebellum posterior lobe (Figure 6).

The cerebellum posterior lobe lies between the cerebellar fissure and the posterolateral fissure and occupies the greater part of the cerebellum in human beings. The function of the cerebellum posterior lobe is to influence the initiation, planning, and coordination of movement and determine the strength, direction, and scope of the movement [22, 23]. In addition, studies have consistently revealed that the cerebellum posterior lobe is also responsible for nonmotor functions [24].

The role of the cerebellum posterior lobe in eye movement control and adaptation is well known [25]. When performing microstimulations on the cerebellums of macaque monkeys, Fujikado and Noda [26] found that the cerebellum posterior lobe was responsible for saccadic eye movements. In another previous study, Lisberger [27] used the concept of internal models, which provided a conceptual structure, to help in understanding how the cerebellum controls eye movement. In addition, Colnaqhi and her associates [28] applied transcranial magnetic stimulation to test cerebellar control of reflexive and voluntary eye movements, proving that the cerebellum posterior lobe plays a key role in reflexive and voluntary eye movements. Furthermore, in research on concomitant strabismus patients, the mean diffusivity values in bilateral posterior cerebellar lobes were found, using digital transducer interface technology, to be significantly decreased [29]. In addition, Tan et al. [20] found that the DC value of the right posterior cerebellar lobe was decreased in patients with concomitant strabismus, indicating that dysfunction of the posterior cerebellar lobe was related to abnormal coordination of the extraocular muscles. All these facts prove that the cerebellum posterior lobe is closely associated with eye movement regulation. Consistent with these findings, we demonstrated decreased DC values in the cerebellum posterior lobe in this study; this finding might reflect the reduction of the role and position of the cerebellum posterior lobe in the whole-brain network in patients with EOPH. Furthermore, we speculatively suggest that the changed functional connectivity in the cerebellum posterior lobe might be associated with eye movement abnormalities in patients with EOPH.

However, the cerebellum posterior lobe also plays a key role in emotion [30, 31]. Lou et al. [32] found that the DC value in the cerebellum posterior lobe was decreased in depressed patients. Therefore, it was speculated that dysfunction of the cerebellar posterior lobe might be related to depression. In another study, patients with obstructive sleep apnea exhibited complex and abnormal DC values in the cerebellum and other brain areas at rest [33]. It has been reported that obstructive sleep apnea patients also experience depression and anxiety [34]. Thus, the brain activity change in the cerebellum posterior lobe might also be a basis of anxiety and depression. Furthermore, Göbel et al. [35] reported that experimental thyrotoxicity could lead to an increase in gray matter volumes in the left and right cerebellum posterior lobe. Moreover, the increase in gray matter volumes in the cerebellum posterior lobe was thought to be compensatory for depression, working memory, and movement coordination. In this study, we used the Hospital Anxiety and Depression Scale to assess the anxiety and depression status of participants. Our results showed that patients with EOPH had higher anxiety and depression scores than the control group. In addition, anxiety, and depression scores were negatively correlated with DC values of the cerebellum posterior lobe. It is likely that the alteration of brain network centrality is also associated with impairment of the emotion procession in patients with EOPH.

The AUC is often utilized to reflect diagnostic accuracy. The ROC result revealed that the AUC of the cerebellum posterior lobe was 1.000, which suggested that there were significant differences in DC values between the EOPH and control groups. Consequently, we inferred that the DC value of the cerebellum posterior lobe might be a potential diagnostic marker for patients with EOPH.

Our research still has some limitations. First of all, our study population is not very large. More participants are needed to further verify the results. Furthermore, the molecular mechanism between the variation of cerebellum posterior lobe activity and exophthalmos in patients with hyperthyroidism needs further investigation.

## 5. Conclusion

To sum up, this study demonstrated that patients with EOPH had abnormal networks in their cerebellum posterior lobe, which indicates that the cerebellum posterior lobe is associated with exophthalmos and provides insight into the neural variation in patients with EOPH.

## Figures and Tables

**Table 1:** Demographics and behavioral results of the EOPH group and control group.

Condition	EOPH	Control group	*t*	*p* value
Male/female	8/12	8/12	N/A	>0.99
Age (years)	54.22 ± 6.88	53.91 ± 6.71	0.129	0.916
Weight (kg)	56.99 ± 6.32	58.92 ± 7.42	0.091	0.817
Handedness	20R	20R	N/A	>0.99
Duration of hyperthyroidism (yrs)	11.32 ± 5.21	12.69 ± 6.17	0.072	0.046
Best-corrected Va in right eye	0.89 ± 0.12	1.01 ± 0.17	−0.492	0.009
Best-corrected Va in left eye	0.92 ± 0.16	1.03 ± 0.13	−0.385	0.012
IOP-R (mmHG)	20.77 ± 3.91	19.14 ± 1.89	0.092	0.782
IOP-L (mmHG)	19.89 ± 4.12	20.91 ± 2.21	0.088	0.826
AS	6.05 ± 1.15	4.20 ± 0.95	5.555	<0.001
DS	5.85 ± 1.09	3.55 ± 0.76	7.746	<0.001

Notes: independent *t*-tests comparing the two groups (*p* < 0.05 represented statistically significant differences). EOPH, exophthalmos of primary hyperthyroidism; N/A, not applicable; Va, visual acuity; IOP, intraocular pressure; R, right; L, left; AS, anxiety scores; DS, depression scores.

**Table 2:** Significant difference in DC values in the cerebellum posterior lobe between EOPH group and control group.

EOPH patients and control groups				MNI coordinates		
Brain areas	BA	*T* values	Peak voxels	*x*	*y*	*z*
Cerebellum posterior lobe		55.6227	93	−15	−84	−27

Notes: the statistical threshold was set at the voxel level with *p* < 0.05 for multiple comparisons using the Gaussian Random Field theory (*z* > 2.3; *p* < 0.01; cluster >40 voxels; AlphaSim corrected). DC, degree centrality; EOPH, exophthalmos of primary hyperthyroidism; MNI, Montreal Neurological Institute; BA, Brodmann area.

## Data Availability

The data used to support the findings of this study are available from the corresponding author upon request.
